# Supplementary material for: Linking belief in a just world and psychological capital to psychological basic needs satisfaction and mental health of young adults living with HIV: A comparative analysis
Source: PLOS Ment Health. 2024 Sep 17;1(4):e0000055. doi: 10.1371/journal.pmen.0000055 (PMC12798245; doi:10.1371/journal.pmen.0000055)
Supplement: Attachment — Submitted filename: Response to Comments - 26.07.2024.docx [file pmen.0000055.s002.docx]

**Response to Comments (Manuscript ID: PMEN-D-24-00143)**

Dear Editors and reviewers,

We glad for the opportunity to revise and resubmit my paper, “Linking Belief in a Just World and Psychological Capital to Psychological Basic Needs Satisfaction and Mental Health of Young Adults Living with HIV: A Comparative Analysis.”

We have made an effort to address all the comments. The responses to the comments are detailed in the table below.

| **No.** | **Comment** | **Response** |
| --- | --- | --- |
| ***Editor comments*** | | |
| 1 | We have suggested that we could re-consider this paper after you make major revisions which are outlined by Reviewers 1 and 2. I agree with both reviewers that you have somewhat lost the wood for the trees and greater clarity required in results and discussion while avoiding extraneous detail. In particular, look at writing in ways that are clear and succinct. For example - these two sentences in the conclusion are somewhat repetitive in concept and you could give a hard edit throughout the document to use a more active tense and journalistic brisk style. Please edit the whole manuscript with this in mind. | We have made efforts to address all the issues raised by the reviewers. We have attempted to improve the language and the flow of the paper. In some parts, we have removed unnecessary details to make the paper more precise. |
| ***Reviewer 1*** | | |
| 1. | Streamline the Introduction: The current introduction is extensive and repetitive. Please condense this section to succinctly cover the scientific background and rationale. Articulate the study's specific objectives and hypotheses clearly and concisely, focusing solely on content that directly supports the study's goals. | The introduction section has been rewritten to provide more conceptual clarity and remove repetitions and other redundancies. |
| 2. | Clarification of Key Terms: Define critical terms such as "young adult" in the abstract or title to remove ambiguity. Also, specify the age range considered for young adults early in the introduction to provide a clear demographic context. | This suggestion has been implemented. |
| 3. | Address Misconceptions Around BJW: It is vital to discuss potential negative perceptions of BJW among people with HIV, particularly concerning self-blame and guilt. Clarify why BJW should not be construed negatively and its implications on self-perception among HIV-positive individuals. | The misconceptions and the negative implications of BJW have been included in the theory and discussion sections. Page 9, paragraph 2. Also see the discussion section - pages 25 - 26 |
| 4. | Review and Justify Sample Size Calculations: Reevaluate the sample size requirements based on the specified effect size, power, and number of predictors. Justify any deviations from typical sample size requirements or address potential limitations if the current sample is underpowered. Discrepancies in the sample size calculations as per the parameters provided were noted; please rectify or clarify these figures. | The sample size calculations have been updated. See page 13 |
| 5. | Enhance Comparative Analysis: Introduce a table detailing the baseline characteristics of both the YALWH and control groups (university students) to ensure comparability. Include variables such as age, occupation, marital status, and socioeconomic status, which are crucial for identifying potential confounders. | This is a pertinent observation. However, we did not collect information on occupational status, marital status, and other socio-economic indicators. We only collected data on age and gender. We have included this issue among the limitations on page 28. |
| 6. | Specify and Elaborate on Regression Models: Ensure that your regression models are correctly specified. Justify the choice and treatment of all covariates, considering potential confounders like socioeconomic status or duration of HIV diagnosis. Provide a detailed explanation or a step-by-step breakdown of complex statistical methods like serial mediation analysis, possibly in supplementary materials, to enhance replicability. | The analytical procedure has been adjusted to provide more clarity. The results section has also been completely rewritten to offer more clarity. |
| 7. | Explore Interaction Effects: Consider adding interaction terms in your analyses to examine how BJW impacts may vary by demographic factors such as age and educational level within your sample groups. | This is an important suggestion. We explored this possibility. However, given that our model involves a double mediation with one of the mediators and outcome variables having two dimensions, the regression models became complex. |
| 8. | Rationalize Use of Likert Scales: Provide a rationale for treating Likert scale responses as interval data. Discuss the methodological implications of this decision and ensure alignment with current psychological research practices. | The rationale for using the Likert scale has been added to the measures section – Page 14. |
| 9. | Clarify Gender Coding: Explain the rationale behind coding gender as a numerical variable and discuss how this approach affects your analysis, ensuring methodological soundness. | The gender coding has been revised to dummy coding. This is useful for dichotomous variables such as gender. |
| 10. | Improve Table Clarity and Relevance: Enhance the clarity and utility of all tables in the manuscript. Each table should clearly support and justify its inclusion through direct relevance to the study's findings. | We have removed two tables relating to the total sample. We believe the tables relating to the two samples take care of the information in the deleted tables. |
| 11. | Justify Control Group Selection: Provide detailed reasoning for using university students as a control group. Discuss their comparability to the YALWH group in terms of educational, geographic, and potentially religious or spiritual affiliations. | The student sample was used as a control group because it is a population of young adults. However, there are important differences between the two groups. Unfortunately, we did not collect data on aspects such as participants' occupational status or religion. We have pointed this out among the limitations on page 28. |
| 12. | Expand Theoretical Context in Discussion: Broaden the discussion section to link your findings explicitly with wider psychological theories beyond BJW and psychological capital, such as theories of stigma and identity, to deepen the interpretation of how BJW impacts mental health among YALWH. | We have made major adjustments in the discussion section, making references to stigma literature and the self-determination theory. |
| 13. | Functional Data Link: The link provided for accessing the data is nonfunctional. Please provide a working link to ensure accessibility and transparency of your data. | The data has been uploaded as a supplementary file. |
|  |  |  |
| ***Reviewer 2*** | | |
| 1. | First, and very importantly, authors are studying several constructs (belief in a just world, psychological capital, mental distress, etc.) which we know that are influenced by individual socio-demographic characteristics. And about this, we only know that the group of students had an age average of 24.74 lower than the group of YALWH (of 30.35, SD=5.98), that the student group were university students and that the participants were recruited from routine HIV/AIDS clinic visits. What is the educational status of the group of YALWH? What is their occupational status (probably, most of them have jobs; if yes, what type of occupation do they have)? Because of the importance of individual socio-demographic variables for the variables under study, a detailed description of the 2 groups of participants if fundamental. | This is a pertinent issue. Indeed, there are variations in the demographics of the two samples.  Unfortunately, we did not collect information regarding educational attainment and occupational status. This makes the comparison between the two groups difficult.  We have pointed out this issue among the limitations of the study on page 28. |
| 2. | Second, the Measures section is insufficient to understand the meaning of the results. No information is described about the language of the instruments: were the items in English? If yes, how was the fluency of the participants in the language? Besides, how was the process of adaptation of the instruments to the Uganda population? No examples of items are provided, so what they are measuring is not clear. Curiously, all the measures performed worse in the YALWH than in the students group. For example, in the mental health scales, α = .75 for the total sample, .84 for the student sample, and .65 for the sample; meaning a marked difference of .20 in reliability between the two groups. Naturally, this raises important concerns about the validity of the results. | The measures section has been improved. The details of the language used and sample items have been included.  By the time we received the reviewer comments, we had managed to collect more data from the YALWH sample, which has been added the analysis. Consequently, the reliabilities of the measures in this sample have improved.  We have included the use of students as a comparable sample in the limitations section on page 28. |
| 3. | Particularly worrying is the case of the variable of “Developmental outcomes”. Authors describe “the attainment of the three developmental tasks was measured using the basic psychological need satisfaction and need frustration questionnaire”. Developmental outcomes are not equivalent to Basic Psychological Needs Satisfaction and Need Frustration. This questionnaire captures individuals Satisfaction and Frustration with 3 basic psychological needs of Relatedness, Autonomy and Competence. Although authors describe the Self-determination Theory in the Introduction, the equivalence that the authors make between Developmental outcomes, “the attainment of three developmental tasks” and Satisfaction/ Frustration with basic psychological needs is misleading. Again, authors make “developmental outcomes”, “attainment of developmental tasks” and “Safisfaction/ Frustration with basic psychological needs”, equivalent, which is not accurate and is misleading. | This is an important observation, and we have taken it seriously. Indeed, measuring developmental outcomes with a measure of basic psychological needs introduced conceptual ambiguity to the paper. We have, therefore, used the basic psychological needs instead of developmental tasks. This adjustment is inline with the measure and theory used, hence providing better conceptual clarity. |
| 4. | Third, the understanding of the relations amongst the variables in the 2 groups would benefit from an integration into a single model. For example, did the authors considered running a mediation moderated model (in which the variable “group” is included as moderator of the relationships amongst the variables? | This is another wonderful suggestion. We attempted a moderated mediation. However, since we are interested in the serial mediation with both psychological capital and satisfaction of basic psychological needs, the moderated mediation became a complex model. |
| 5. | Finally, the authors identify the variable of “Psychological Wellbeing” as the outcome variable. However, in the measures section, there is no reference to the variable of “Psychological wellbeing”. I imagine that that authors are estimating the variable “Psychological Wellbeing” using the two indicators of Mental Health. However, no information is presented about this in the Methods. This is not a minor issue. In fact, I worry that authors use concepts as if they were equivalent (in their meaning, operationalization, and measurement). The manuscript lacks consistency in what concerns the use of concepts and constructs, and this requires particular attention and revision. | The paper focuses on mental health; factorized as flourishing and distress.  We have made corrections where this had been misrepresented as psychological well-being. |
